# Psychological Aspects of Cosmetic Surgery among Females: A Media Literacy Training Intervention

**DOI:** 10.5539/gjhs.v8n2p35

**Published:** 2015-06-01

**Authors:** Zahra Khazir, Tahereh Dehdari, Mahmood Mahmoodi Majdabad, Said Pournaghash Tehrani

**Affiliations:** 1 School of Health, Tehran University of Medical Sciences, Tehran, Iran; 2 School of Health, Iran University of Medical Sciences, Tehran, Iran; 3 School of Psychology, University of Tehran, Tehran, Iran

**Keywords:** attitude, elective cosmetic surgery, body dissatisfaction, self-esteem, body dysmorphic disorder, media literacy education, Iran

## Abstract

**Introduction:**

The present study examined the favorable attitude of a sample of female university students regarding elective cosmetic surgery, body dysmorphic disorder, self-esteem and body dissatisfaction following a media literacy training intervention.

**Methods:**

This study was a quasi-experimental type. The study sample included 140 female university students who were allocated to either the intervention (n=70) or the control group (n=70). Attitude toward cosmetic surgery, body dysmorphic disorder, self-esteem and, body satisfaction was measured in both groups before the intervention and 4 weeks later. Four media literacy training sessions were conducted over 4 weeks for the intervention group. The data was analyzed through analysis of covariance, student’s paired-samples t test, and Pearson correlation.

**Results:**

Our findings showed that favorable attitude, body dysmorphic disorder and body dissatisfaction scores were significantly lower (p<0.05) in the intervention group than the control group. Furthermore, self-esteem score increased significantly in the intervention group.

**Conclusions:**

Our results underscores the importance of media literacy intervention in decreasing female’s favorable attitude towards elective cosmetic surgery, body dysmorphic disorder and body dissatisfaction as well as increasing self-esteem.

## 1. Introduction

The several past decades have witnessed an increase in the number of teenagers and young people choosing to have cosmetic surgery (Mianroodi, Eslami, & Khanjani, 2012). According to a report by American Society for Aesthetic Plastic Surgery (ASAPS), about 11 million surgical and nonsurgical cosmetic procedures were performed in the United States in 2013. Total number of cosmetic surgical procedures increased by 6.5% between 2012 and 2013. The rate of cosmetic procedures is rising more rapidly among women (The American Society for Aesthetic Plastic Surgery, 2014). Little information is available regarding the total number of cosmetic procedures performed annually in Iran. However, literature review shows that cosmetic surgery especially rhinoplasty has been the most prevalent type of all in Iran (Mianroodi, Eslami, & Khanjani, 2012, 2012; Farshidfar, Dastjerdi, & Shahabizadeh, 2013).

Several psychological factors can impact the willingness of undergoing cosmetic surgery including body dysmorphic disorder, attitude, low self-esteem and body dissatisfaction (Farshidfar, Dastjerdi, & Shahabizadeh, 2013; Henderson-King & Henderson-King, 2005; Sarwer & Crerand, 2004; Sarweq, Wadden, Pertschuk, & Whitaker, 1998). Body dysmorphic disorder, as one of the most common body image disorders, is defined as any distressing and/or impairing preoccupation with a nonexistent or slight defect in physical appearance (Honigman, Phillips, & Castle, 2004). Self-esteem is defined as the person’s emotional evaluation of his/her own worth (Hewitt, 2009). In addition, body dissatisfaction is defined as the person’s negative feelings towards a certain body part, or one’s body shape or weight (Grogan, 1999). It is noteworthy that mass media, through portraying the social and cultural standards of the beauty and ideal body image, has a direct influence on the development of these psychological aspects thereby increasing the demand for different types of plastic surgery (Sarwer, Magee, & Crerand, 2004; Agliata & Tantleff-Dunn, 2004; Tucci & Peters, 2008; Groesz, Levine, & Mumen, 2002; Champion, & Furnham, 1999). The media’s misleading advertisement of unrealistic standards of beauty as well as underestimating the dangers of cosmetic surgical procedures and its early and future side effects (e.g., hemorrhage, skin necrosis, synechiae formation, polly beak nasal deformity, oleogranuloma, scar hypertrophy, perinasal trauma, pulmonary thromboembolism and death (Grazer, & de Jong, 2000; Fernandes, 2008; Gandomi, Arzanghi, Sharifi, Tabibi, & Alipoor, 2011) create a favorable environment where consumers have false and unrealistic expectations of cosmetic surgery outcomes. Empowering individuals to critique message media will effectively reduce and even eliminate these expectations (Yamamiya, Cash, Melnyk, Posavac, & Posavac, 2005). Literature showed that conducting interventions such as media literacy education for improving individuals^,^ critical thinking skills against media messages are needed (Levine, Piran, & Stoddard, 1999; Bergsma & Carney, 2008). Media literacy has been defined as the ability of individuals to identify, analyze, evaluate, and communicate messages in the media (Aufderheide, 1993). The aim of media literacy education is to inform the youth that the media is in the business of selling products (Eagle, 2007). Even though media literacy training researches are still limited, several studies have shown its effectiveness in addressing a variety of psychological aspects such as reducing weight concerns and body dissatisfaction, better internalization of body stereotypes, and improving self-esteem (Grogan, 2010; Kusel, 1999; Wade, Davidson, & O’Dea, 2003). Given the high prevalence of cosmetic surgery in Iran, there is a need for development of preventive interventions such as media literacy education. As such, the present study was designed to determine the favorable attitude of a sample of female university students towards elective cosmetic surgery as well as body dysmorphic disorder, self-esteem, and body dissatisfaction following a media literacy training intervention.

## 2. Methods

### 2.1 Participants and Setting

In this quasi-experimental study, two female dormitories of Tehran University of Medical Sciences were selected trough random sampling method. To reduce possible contamination of the control group with the intervention group, students of one dormitory were assigned into the control and the other dormitory into the intervention group. Then, given the calculated sample size, seventy female college students were selected by systematic random sampling method from each dormitory. This study was conducted between June 2012 and October 2013 in Tehran, Iran, and was approved by the ethics committee of Iran University of Medical Sciences. Participants were informed about the objectives of the study and a written consent was obtained from them. All of the eligible students willingly agreed to take part in the study.

### 2.2 Sample Size Calculation

Sample size was determined based on the estimated mean differences between the intervention and control group on pre-assessment to post assessment changes on body image concern (effect size=2) and standard deviation (S=4.3) in a study by McVey et al (McVey, Davis, Tweed, & Shaw, 2004) and a power level of 80% at a significance level of 0.05. The Pocock formula used to calculate the sample size *[n=2(z_1-α/2_+z_1-β_)^2^S^2^/(µ_0_-µ_1_)^2^]*. The final sample size was 140 participants with 70 in each group (control and intervention).

### 2.3 Procedure

Two trained research assistants not involved in the intervention, four times a week at the two dormitories distributed the questionnaires among the participants prior to the intervention. The questionnaires were completed by the students in approximately 25-30 minutes. In the next stage, a media literacy education intervention was developed for the students of the intervention group while the participants in the control group received no intervention. Considering that intervention program was implemented by two trained experts in health education and psychology as well as one medical student. Finally, the two groups were followed up for 4 weeks after the intervention.

### 2.4 Study Instrument and Measures

#### 2.4.1 Demographic Characteristics

The demographic data of the participants including age, body mass index (BMI), marital status, parent’s education level, occupation, and family size were collected.

#### 2.4.2 Attitude toward Cosmetic Surgery

This variable was measured by means of “Acceptance of Cosmetic Surgery Scale” which includes 15 items on a 7-point Likert-type scale ranging from 1 = strongly disagree to 7 = strongly agree. The scale was developed by Henderson-King and Henderson-King (Henderson-King & Henderson-King, 2005) and its validity and reliability has been approved previously. This scale evaluates three dimensions including individual attitude toward cosmetic surgery-related benefits (sample item: “If cosmetic surgery could make someone happier with the way they look, then they should try it”), social motivations for cosmetic surgery practice (sample item: “If it would benefit my career, I would think about having plastic surgery”), and the likelihood of considering cosmetic surgery by an individual (sample item: “If I could have a surgical procedure done for free, I would consider trying cosmetic surgery”). A higher score in attitude indicates that the person is more predisposed to undergoing cosmetic surgery. In the present study, Cronbach’s α for intrapersonal, social, and consider subscales were 0.78, 0.90, and 0.79, respectively.

#### 2.4.3 Body Dissatisfaction

In this study, “Body Satisfaction Scale” (BSS) was used to assess satisfaction/dissatisfaction towards 16 body parts, half involving the body (below the head) and the other half involving the head (above the neck). The scale was developed by Slade et al (Slade, Dewey, Newton, Brodie, & Kiemle, 1990). Sixteen items of this scale are measured on a Likert-type scale ranging from 0 (strong satisfaction) to 7 (strong dissatisfaction). The psychometric quality of BSS has been reported to be satisfactory (Bergsma & Carney, 2008). Higher scores indicate higher body dissatisfaction. The Cronbach’s α for this scale was 0.90.

#### 2.4.4 Body Dysmorphic Disorder

This variable was measured using “Body Image Concern Inventory” (BICI). This self-administered measure was developed by Ghadakzadeh et al. (Ghadakzadeh, Ghazipour, Khajeddin, Karimian, & Borhani, 2011) for assessing body dysmorphic disorder among Iranians seeking rhinoplasty. The validity and reliability of this scale has been reported to be acceptable. Nineteen items of this scale are measured on a Likert-type scale ranging from 1 (never) to 5 (always); (sample items: “If cosmetic surgery has a positive effect on my future occupation, I would consider having it” or “People who are not quite satisfied with their appearance, should think about plastic surgery as a solution”). Higher total scores represent more dysmorphic concerns. The Cronbach’s α for this scale was 0.80.

#### 2.4.5 Self-Esteem

Self-esteem was measured by the 10-item “Rosenberg’s Self-Esteem Scale” (Rosenberg’s SES). The items of this scale are measured on a Likert-type scale ranging from 0 (strongly disagree) to 3 (strongly agree); (sample item:”I take a positive attitude towards myself”). Items 3, 5, 8, and 9 are reversely scored. Shapurian et al (Shapurian, Hojat, & Nayerahmadi, 1987) adapted this scale to use in Iran. A higher sum of scores shows higher self-esteem. The Cronbach’s α for this scale was 0.80.

### 2.5 Media Literacy Program

The media literacy intervention was developed based on previous researches in terms of media literacy education (Coughlin & Kalodner, 2006; Hensley Choate, 2007; National Association for Media Literacy Education, 2007; Halliwell, Easun, & Harcourt, 2011). The media literacy education intervention for the participants in the intervention group included four 40-60 minute training sessions over 4 weeks. There was a 7-day interval between the sessions. Students were divided into seven groups of 10 people. The sessions were designed to be interactive and discussion-oriented. The content of sessions is summarized in Table 1.

**Table 1:** Outline of the media literacy intervention

	Objectives	Materials & Activities
***Session 1:*** (40 minutes)	a) to provide elective cosmetic surgery statistics in Iran and the world, b) to explain and discuss body ideals and provide a history of the standards of beauty among Iranian women, c) to explain dangers of elective cosmetic surgery procedures and economic motivations of plastic surgeons and d) to explain women’s beliefs about cosmetic surgery and their expectations of such procedures	-Slides showing the prevalence of cosmetic surgery in Iran and the world -Video about females who had elective cosmetic surgery with complications such as facial nerve injury, unfavorable scar formation, skin loss, etc and group discussion -Slides showing the changes in the standards of beauty among Iranian women -individual brainstorming regarding beliefs and expectations and negative and positive experiences regarding elective cosmetic surgery.
***Session 2:*** (60 minutes)	a) to explain the influence of the media in creating false and unrealistic expectations about elective cosmetic surgery and dissatisfaction about one^’^s physical appearance, b) to explain persuasive marketing and misleading advertising (e.g. unrealistic beauty images) of media, c) to inform about the techniques used by the media (e.g. airbrushing and computer graphics) to create ideal body images and models (the media’s manipulation of images), and d) to introduce cognitive strategies (critical analysis and expression skills) for challenging the messages they receive from the mass media and resisting the advertising pressure for cosmetic surgery practice.	-Slides of misleading advertising in various media -Small group work: habits of inquiry, analyze, evaluate, and use media messages -Small group work: selection of advertisements about cosmetic surgery (what are they trying to tell? are they realistic?) -An instructional booklet about the mentioned objectives.
***Session 3:*** (60 minutes)	a) to review introduced cognitive strategies in session 2, b) to provide statistics about the prevalence of body dissatisfaction and body dysmorphic disorder in cosmetic surgery candidates, c) to explain the process by which the media leads to disturbances in body image, self-esteem and body satisfaction, d) to explain body comparison and its negative consequences, and e) training thought stopping and relaxation methods to extinguish distress about appearance (Rosen, Reiter, & Orosan, 1995)^.^	-Small groups: what can we do to feel positive about our body? -Small groups: ask the participants to identify and then discuss functional aspects of their bodies and positive qualities that are not associated with appearance -Showing short ‘Evolution’ video produced by Dove in avoiding body comparison with media images (Richardson & Paxton, 2010). -An instructional pamphlet about the method of thought stopping practice -An audio CD of Progressive Muscular Relaxation.
***Session 4:*** (50 minutes)	a) to review strategies introduced in session 3, b) to provide information about the role of self-esteem in preventing elective cosmetic surgery, and c) to introduce strategies focused on enhancing self-esteem.	-Slides regarding six strategies introduced by WHO for increasing general self-esteem (including: 1) take a Self-Esteem Inventory, 2) set realistic expectations, 3) set aside perfection and grab a hold of accomplishments and mistakes, 4) explore yourself 5) be willing to adjust your own self-image, and 6) stop comparing yourself to others) (Groho, 2014).

### 2.6 Statistical Analysis

The data were analyzed by SPSS software package (version 18.0, SPSS, Inc., Chicago, IL, USA). The homogeneity of demographic variables of the two groups was analyzed by independent-samples *t* tests and Chi-square. Date normality was tested using Kolmogorov-Smirnov test. The differences in the total mean scores of attitude; body dysmorphic disorder, body dissatisfaction, and self-esteem before and after the intervention were tested by means of Student’s paired-samples t-test. Differences in the total mean scores of attitude, body dysmorphic disorder, body dissatisfaction, and self-esteem between the groups after the intervention were also tested using Analysis of Covariance (ANCOVA). Differences in the total mean scores of these variables between the groups before the intervention were also tested using independent-samples *t* tests. Correlations among attitude, body dysmorphic disorder, body dissatisfaction, and self-esteem scores were determined by Pearson correlation coefficient. The data were reported as mean±SD. P<0.05 was considered significant.

## 3. Results

Table 2 shows the demographic characteristics of the female university students in the two groups. No significant

**Table 2:** Descriptive statistics of characteristics of university students in the two groups

	Intervention group (n=70)		Control group (n=70)	

	N (%)	Mean (± SD)	N (%)	Mean (± SD)
Age		21.42±1.73		21.62±21.20
Marital status				
Single	65 (92.9)		65 (92.9)	
Married	5 (7.1)		5 (7.1)	
Divorced				
Body Mass Index (BMI)		21.10±2.71		21.32±2.64
Family size		2.71±1.70		2.72±1.73
Father’s education				
Illiterate	2 (2.9)		0 (0)	
≤12^th^ (grade)	27 (38.5)		40 (57.1)	
>12^th^(grade)	41 (58.6)		30 (42.9)	
Mother’s education				
Illiterate	5 (7.1)		4 (5.7)	
≤12^th^ (grade)	34 (48.5)		49(70)	
>12^th^(grade)	31 (44.3)		17 (24.3)	
Mother’s occupation				
Self-employed	1 (1.4)		2 (2.9)	
Employee	20 (28.6)		13 (18.6)	
Housekeeper	43 (61.4)		51 (72.9)	
Retired	6 (8.6)		4 (5.7)	
Father^’^s occupation				
Self-employed	20 (28.6)		21(30)	
Employee	25 (35.7)		30 (42.9)	
Retired	25 (35.7)		19 (27.2)	

differences were found in demographic characteristics, and dependent variables including attitude, body dysmorphic disorder, body dissatisfaction, and self-esteem at baseline.

Mean scores of attitude, body dysmorphic disorder, body dissatisfaction, and self-esteem on pre- and post-assessment are shown in Table 3 for both groups. The results of the paired samples *t*-test showed that there was a significant reduction in body dissatisfaction score in both groups after the intervention. In addition, significant improvements were observed in self-esteem score in the two groups. Results indicated that the intervention group reported a significantly lower body dysmorphic disorder, and favorable attitude toward elective cosmetic surgery compared to the after the intervention.

**Table 3:** Comparison of favorable attitude, body dysmorphic disorder, body dissatisfaction, and self-esteem in the two groups before and after the intervention

	Intervention group (n=70)						Control group (n=70)					

	Before intervention		After intervention				Before intervention		After intervention			

	Mean	*SD*	Mean	*SD*	*t*	*p value*	Mean	*SD*	Mean	*SD*	*t*	*P value*
Favorable attitude	61.53	17.44	43.76	16.55	6.68	0.001^*^	62.04	22.03	59.03	21.14	1.53	0.13
Body dysmorphic disorder	45.13	13.65	28.39	10.69	-6.04	0.001^*^	43.67	17.92	41.10	13.38	-1.55	0.12
Body dissatisfaction	105.46	13.70	87.92	24.03	8.52	0.001^*^	99.29	12	98.55	12.26	2.22	0.03^*^
Self-esteem	32.91	4.77	36.07	3.59	-4.67	0.001^*^	33.65	5.66	34.95	4.27	-3.49	0.001^*^

Mean values were significantly different from those before the intervention (paired-samples t test):

* P<0.05.

ANCOVA conducted to make over-group comparisons, also revealed significant outcomes. As shown in table 4, the intervention group reported significantly lower body dissatisfaction, favorable attitude and body dysmorphic disorder scores following the intervention as compared to the control group. Moreover, self-esteem in the intervention group was significantly higher than the control group after the intervention.

**Table 4:** Comparison of favorable attitude, body dysmorphic disorder, body dissatisfaction, and self-esteem between the two groups after the intervention

	Intervention group (n=70)		Control group (n=70)		

	Mean	*SD*	Mean	*SD*	*P value*
Favorable attitude	43.76	16.55	59.03	21.14	0.0001^*^
Body dysmorphic disorder	28.39	10.69	41.10	13.38	0.0001^*^
Body dissatisfaction	87.92	24.03	98.55	12.26	0.0001^*^
Self-esteem	36.07	3.59	34.95	4.27	0.03^*^

Mean values were significantly different from those of the control group after the intervention (analysis of covariance):

* P<0.05.

Correlations among attitude, body dysmorphic disorder, body dissatisfaction, and self-esteem variables in the two groups before and after the intervention are presented in Table 5. Results showed that in both groups, there was significant positive association between favorable attitude toward cosmetic surgery and body dissatisfaction (p<0.05). In addition, there was inverse significant association between body dissatisfaction or body dysmorphic disorder and self-esteem in the two groups before and after the intervention (p<0.05). No significant association was observed between favorable attitude towards cosmetic surgery and self-esteem in the two groups (p>0.05).

**Table 5:** Correlation among favorable attitude toward cosmetic surgery, body dysmorphic disorder, body dissatisfaction and self-esteem scores in the two groups before and after the intervention

	Intervention group (n=70)								Control group (n=70)							

	Pre-test				Post-test				Pre-test				Post-test			

	1	2	3	4	1	2	3	4	1	2	3	4	1	2	3	4
1. Favorable attitude	1				1				1				1			
2. Body dissatisfaction	0.29*	1			0.59*	1			0.46*	1			0.41*	1		
3. Body dysmorpc disorder	-0.06	-0.7*	1		-0.44*	-0.25*	1		0.34	-0.46*	1		-0.31*	-0.32*	1	
4. Self-esteem	-0.03	-0.09^*^	0.01^*^	1	-0.22	-0.33*	0.41*	1	0.09	0.37*	0.33*	1	-0.18	-0.40*	0.41*	1

## 4. Discussion

The results of the present study indicated that, following the intervention, the favorable cosmetic surgery attitude in the intervention group was significantly lower than the control group, findings in line with those of Jeong et al. (2012) and Rabak-Wagener et al. (1998) (Jeong, Cho, & Hwang, 2012; Rabak-Wagener, Eickhoff-Shemek, & Kelly-Vance, 1998). We found that attitude was positively related to body dysmorphic disorder (Table 5). Body image concern may motivate the pursuit of cosmetic medical treatments in individuals (Sarwer & Crerand, 2004; Sarwer, 2003). Sarwer et al. (Sarwer, 2005) showed that the attitude toward cosmetic surgery was significantly and positively related to body dysmorphic disorder among women. Von Soest et al. (2006) also demonstrated that body image concern was one of the strongest predictors of the cosmetic surgery motivation among Norwegian women aged 22 to 55 years (Von Soest, Kvalem, Skolleborg, & Roald, 2006). In agreement with findings of these studies, results of the present study showed that body dysmorphic disorder variable, as a predictor of attitude toward elective cosmetic surgery, should be considered in developing interventions aimed at modifying women’s attitude and reducing possibility of undertaking cosmetic surgery among them.

The effect of the media literacy intervention on the mean score of body dysmorphic disorder in the intervention group was, with a significant decrease from a mean of 45.13 (SD 13.65) at baseline to 28.39 (SD 10.69) after the intervention, which agrees with other studies that showed psycho-educational media-literacy interventions may reduce the effects of media exposure in body dysmorphic disorder among women (Yamamiya, Cash, Melnyk, Posavac, & Posavac, 2005; Wolf-Bloom, 1998). It is worth noting that although exposure to media images negatively affects the body image concern and mood of females, not all of them are equally susceptible to these described effects (Yamamiya, Cash, Melnyk, Posavac, & Posavac, 2005). Variables such as psychological function, parental feedback, attractiveness and beauty-ideal internalization may increase the effects of the media on body image concern among women (Tiggemann & McGill, 2004; Schwartz, Phares, Tantleff-Dunn, & Thompson, 1999; Thompson & Heinberg, 1999). However, more research is needed in order to determine other effective methods of reducing body dysmorphic disorder among females (e.g. cognitive behavior therapy). For instance, Rosen et al. (1995) reported that cognitive behavior therapies regarding body image concern significantly decreased body dismorphic disorder symptoms among women (Rosen, Reiter, & Orosan, 1995). The present results also demonstrated that the media literacy intervention was able to reduce the mean score of body dissatisfaction in intervention group. This finding is in agreement with those of Kusel and Wolf-Bloom’s (Kusel, 1995; Wolf-Bloom, 1998). They reported that media literacy training may help increase body satisfaction in girls. It has been also demonstrated by Coughlin and Kalodner that women at high-risk for eating disorders gained lower scores on body dissatisfaction, feeling of ineffectiveness, and internalization of socio-cultural standards of beauty after participating in a media literacy education program (Coughlin & Kalodner, 2006). Since the media can influence one’s self-esteem, body dissatisfaction, and favorable attitude directly (Champion & Furnham, 1999; Slevec & Tiggemann, 2010; Bessenoff, 2006), developing early media literacy training interventions to help females deconstruct advertising and media images becomes of great importance (Clay, Vignoles, & Dittmar, 2005). As a result, modifying policies and regulations on elective cosmetic surgery advertising and marketing messages becomes necessary for increasing the effect of media literacy interventions.

In the current study, self-esteem increased significantly after the intervention as compared to the control group. Kusel and Wolf-Bloom (1998) have emphasized the positive influence of the media literacy program in increasing self-esteem of young girls, which is in agreement with the results of the present study (Kusel, 1999; Wolf-Bloom, 1998).

Although the findings in the present study underscore the effect of media literacy training on psychological aspects of cosmetic surgery among a sample of female college students, it had some limitations. Data were collected from female college students who resided in the dormitories of Tehran University of Medical Sciences. Thus, the findings are not generalizable to other groups of university students (e.g. male university students or those who do not live in the dormitory). Assessing the impact of media literacy intervention within these groups requires further investigation. Also, we examined the short-term impact of media literacy intervention on persuasive psychological factors for having elective cosmetic surgery. More research is needed to investigate whether this effect can last for longer periods of time.

### 4.1 Implication for Future Research

As the prevalence of elective cosmetic surgery among females in various cultures and countries is high (Mianroodi, Eslami, & Khanjani, 2012; The American Society for Aesthetic Plastic Surgery, 2014; Farshidfar, Dastjerdi, & Shahabizadeh, 2013), more research is required to clarify the role of media literacy training in reducing the need for having cosmetic surgery among females from other racial/ethnic groups and cultures, especially in Iran. Most of the interventions performed in the field of media literacy have focused on eating disorders and weight reduction (Coughlin & Kalodner, 2006; Wolf-Bloom, 1998), while to the best of our knowledge; no standard curriculum has been developed for training media literacy in terms of cosmetic surgery. Therefore, it is crucial to develop such standard curriculums to be used in different cultures. Behavior change theories and models, such as conceptual frameworks, can help practitioners realize why people do or do not engage in certain behaviors (National Cancer Institute, 2005; Jackson, 1997), therefore it is suggested that researchers develop and conduct theory-based interventions (e.g. Theory of Reasoned Action) aimed at decreasing women’s desire for undergoing cosmetic surgery.

## 5. Conclusion

Overall, the results of this study underscore the importance of media literacy intervention in decreasing female’s favorable attitude towards elective cosmetic surgery, body dysmorphic disorder and body dissatisfaction as well as increasing self-esteem.
